# Aiding empirical research on the commercial determinants of health: a scoping review of datasets and methods about lobbying

**DOI:** 10.1186/s12961-023-01011-8

**Published:** 2023-06-19

**Authors:** Jennifer Lacy-Nichols, Madalyn Quinn, Katherine Cullerton

**Affiliations:** 1 Centre for Health Policy, Melbourne School of Population and Global Health, The University of Melbourne, Level 5, 207 Bouverie St., Carlton, VIC 3053 Australia; 2 Melbourne School of Population and Global Health, The University of Melbourne, Carlton, Australia; 3 School of Public Health, The University of Queensland, Herston, Australia

**Keywords:** Lobbying, Corporate political activity, Data, Public health, Commercial determinants of health

## Abstract

**Background:**

To support public health researchers and advocates seeking to challenge the influence of powerful commercial actors on health, it is necessary to develop a deeper understanding of corporate political activities. This project explores political science scholarship analysing lobbying to identify new datasets and research methods that can be applied to public health and stimulate further research and advocacy.

**Methods:**

We undertook a systematic scoping review of peer-reviewed and grey literature reports analysing the practice of lobbying. Titles and abstracts of 4533 peer-reviewed and 285 grey literature reports were screened, with 233 peer-reviewed and 280 grey literature reports assessed for eligibility. We used a two-stage process for data extraction. In stage 1, we collected two pieces of information from all included studies: data sources and indicators used to measure lobbying. For the second stage, data extraction was limited to 15 studies that focused on meetings.

**Results:**

The most common indicators used to measure lobbying activity were: registrations of active lobbyists; expenditure on lobbying; meetings; written comments and submissions made to government consultations; bills; and committee participation. A range of different data sources were used to analyse lobbying, including from governments, not-for-profits and commercial sources. All 15 studies analysing lobbyist meetings were from high-income contexts. The studies analysed three key variables: the types of government actors targeted by lobbying; the policies of interest; and the lobbyists and/or their clients. The studies used a range of taxonomies to classify policy issues and the types of actors engaged in lobbying. All studies discussed challenges with accessing and analysing lobbying data.

**Conclusions:**

There is enormous potential for public health research and advocacy concerned with commercial lobbying to learn from political science scholarship. This includes both conceptual frameworks and sources of empirical data. Moreover, the absence of good quality transparency internationally emphasises the importance of advocacy to support policy change to improve the quality of political transparency to make it easier to monitor commercial lobbying.

**Supplementary Information:**

The online version contains supplementary material available at 10.1186/s12961-023-01011-8.

## Introduction

Many of the most effective and equitable policies to reduce and prevent the burden of non-communicable diseases (NCDs) present risks to the profits of powerful corporations. Excise taxes, warning labels, restrictions on marketing and other interventions are designed to reduce the appeal, affordability, and accessibility of harmful products such as tobacco, alcohol and sugary drinks [1]. To successfully implement these policies, we need to understand, anticipate, and challenge the political practices corporations use to undermine and block public health policies.

Public health researchers are increasingly turning their attention to the commercial determinants of health (CDoH)—the ways in which commercial actors drive health and equity [2, 3]. Of course, the CDoH need not be harmful—companies can provide living wages and improve access to health promoting products and services like fresh fruits, education and healthcare. [4] However, much of the CDoH literature has rightly drawn attention to the damaging practices of powerful transnational corporations manufacturing products that harm human health, the environment, human rights and democracy [3]. Examples of these practices include predatory marketing [5], denying scientific evidence [6], polluting communities [7] and blocking public health regulation [8]. This paper focuses on one of the seven key practices through which commercial actors influence health: political practices [3].

A first step towards challenging corporate political practices is developing a far deeper understanding of the nature of corporate political practices. What is the range of political practices used? Which industries or actors use those practices, and which use others? Why do they use those practices? What is the influence of different market or regulatory circumstances on corporate political practices? What patterns can be observed over time? These questions indicate the potential scope of inquiry for future research on corporate political activity. This paper builds on the exhortations for public health advocates and policymakers to understand more deeply the ‘broader trends and patterns’ of corporate political activity [9]. There is a need to develop strategies and tools to systematically monitor corporate political practices as they occur and to enable comparisons across differing companies, industry sectors, levels of government and countries [10-12].

Currently, opportunities to analyse corporate political lobbying are largely determined by the availability and quality of publicly available data. Yet the very nature of the activity, one that often occurs behind closed doors and with varying degrees of formality and informality renders it difficult to define and regulate. This makes for an exceedingly opaque practice and is a significant barrier for researchers. In Australia, for example, the federal, state and territory governments provide lobbyist registers with information about lobby firms and their clients. Yet the registers can obscure more than they reveal: the Northern Territory has no register, no jurisdiction includes ‘in-house’ lobbyists directly employed by companies, and information about lobbyists’ previous government employment is patchy and vague [13-15]. These issues are not unique to Australia. A 2021 survey conducted by the Organisation for Economic Cooperation and Development (OECD) found that only 23 of 41 jurisdictions provided information about lobbying, and that the quality of lobbying transparency and disclosure varied significantly [16]. While governments around the world clearly need to improve transparency and disclosure practices, for public health researchers interested in examining these issues, more sophisticated tools to monitor corporate political practices need to be developed.

To help overcome these challenges, we set out to explore how one political practice—lobbying governments—has been analysed outside the CDoH field. CDoH researchers and public health researchers more generally are relatively new entrants to the study of corporate lobbying and lobbyists, which has long been the domain of political science [17]. It is likely that there are many datasets and methods already in use that could advance public health efforts to monitor corporate lobbying. In our experience, one of the key hurdles is the human resources required to extract, clean and analyse data systematically. For that reason, we were interested in identifying the different methods and tools used to minimise or circumvent the need for laborious manual coding and analysis. Two practical questions guided our approach: (1) Where can we find data about lobbying, and (2) How can we access and analyse this data? To answer these questions, we conducted a systematic scoping review of peer-reviewed and grey literature analysing lobbying activities internationally. While it was not possible for our review to comprehensively document all available data sources or methods, our aim was to exemplify the diversity of possible sources and methods to stimulate further research.

In the following sections, we provide a brief overview of public health research on lobbying, including efforts to monitor corporate lobbying and challenges identified in the literature. We then present our research methods and the findings of our scoping review. In the discussion, we reflect on what practical insights CDoH researchers can acquire from political science scholarship on lobbying and what could be changed from a policy standpoint to make it easier to monitor commercial lobbying.

### Researching and monitoring corporate lobbying

Lobbying is fundamentally about political influence. While lobbying is a legitimate activity, the dominance of business interests has triggered citizens’ concerns of conflicts of interest, undue influence and corruption in government [18]. Although any individual or organisation can lobby governments, in practice, business interests are the most common demographic represented by lobby firms [18]. This is unsurprising, as the financial resources and personal connections that facilitate access to politicians are often held by businesses.

Public health literature conceptualises lobbying in different ways. Frequently, the practice of lobbying is not defined, but rather used generally to refer to different activities to ‘influence’ policymakers. For instance, Miller and Harkins [19] describe lobbying as a strategy to ‘capture’ different arenas of decision making, including the arenas of science, media, civil society and policy. The range of different practices described in the public health literature as lobbying include: meetings with ministers, special advisors and civil servants [20, 21]; corporate philanthropic donations [22]; funding astroturf organisations (industry sponsored groups masquerading as grassroots civil society) [19]; building long-term relationships with key decision makers [23]; engaging with public servants via party conferences and other channels [23]; and written submissions [21]. Lobbying can also encompass political donations, public relations, gifts, the ‘revolving door’ between employment in the public and private sector, participation in advisory groups, the use of think tanks and more [9, 24]. These diverse, and often hidden, activities highlight the practical challenges of documenting and monitoring lobbying in its entirety.

Both the Corporate Political Activity (CPA) Framework and the Policy Dystopia Framework (developed by the tobacco control research group) provide slightly different definitions of lobbying. The CPA framework conceptualises lobbying as part of an ‘Information’ strategy, defined as ‘meetings and correspondence with legislatures/policymakers’ [9]. The framework differentiates between direct and indirect lobbying, where the former is undertaken by tobacco companies themselves, and the latter by organisations less directly affiliated with the tobacco industry, such as business associations, unions or front groups that are used to camouflage tobacco industry interests. In the subsequent Policy Dystopia Framework, lobbying is segmented into strategies of information management and direct involvement/influence, with the later comprising the techniques of access, incentives and threats, actor in legislative processes, and actor in government decision-making [8]. In both frameworks, lobbying is conceptualised as a part of a wider set of political strategies to influence decision-making.

As outlined, one of the challenges for lobbying research and policy making is defining the practice. Lobbying and lobbyists can be defined in different ways (Table 1 lists illustrative examples of diverse definitions). Other terms used to refer to lobbying or those undertaking lobbying include lobbyists, advocacy, interest groups, special interests and influence. Indeed, a 2009 OECD report found that every country surveyed varied in its legal definition of lobbying [25]. This inconsistency makes it challenging to document and analyse the practice of lobbying across countries, to develop robust monitoring systems, and to compare datasets.

**Table 1:** Definitions of lobbying

Definition	Year	Source
Interest representation (lobbying): all activities carried out with the objective of influencing the policy formulation and decision-making processes of the European institutions	2007	European Transparency Initiative [26]
To make deals and influence political processes	2008	World Health Organization [27]
Any contact (written or oral communication, including electronic communication) with lobbying targets for the purpose of influencing the formulation, modification, adoption, or administration of legislation, rules, spending decisions, or any other government program, policy, or position	2013	Sunlight Foundation [28]
The act of lawfully attempting to influence the design, implementation, execution and evaluation of public policies and regulations administered by executive, legislative or judicial public officials at the local, regional or national level	2021	Organisation for Economic Co-operation and Development [16]
Any activity carried out to influence a government or institution’s policies and decisions in favour of a specific cause or outcome. Even when allowed by law, these acts can become distortive if disproportionate levels of influence exist – by companies, associations, organisations and individuals	2022	Transparency International [29]
Any direct or indirect communication with a public official that is made, managed or directed with the purpose of influencing public decision-making	2022	International standards for lobbying regulation [30]

To support systematic monitoring of lobbying and other political practices, public health researchers have developed different conceptual frameworks and taxonomies to classify different political practices. The *Corporate Political Activity* and *Policy Dystopia Frameworks* discussed above are two examples that have been applied to tobacco, alcohol, ultra-processed food, infant formula and other industries to document the extent and range of activities across countries. Other approaches have sought to develop indicators to measure the influence of corporate political practices, such as the *Corporate Permeation Index,* the *Corporate Financial Influence Index* and the *Commercial Determinants of Health Index*, the latter of which includes the number of registered lobbyist as well as gaps in national regulation of lobbyists as indicators for the level of CDoH risk exposure [10, 31, 32].

A frequent conclusion of public health research on lobbying is that data are often difficult to access and incomplete [33]. In the absence of consistent transparency around lobbying, researchers and advocates must balance the desire for detailed information with the need for completeness. In the protocol developed for the *Corporate Financial Influence Index*, the authors reflect on the challenges of finding datasets measuring lobbying transparency with sufficient country coverage, ultimately excluding the lobbying indicator for that reason and focusing on financial influence as opposed to political influence more broadly in the final study [34]. A further challenge for frameworks that measure corporate political activity is that the indicators with the widest coverage often measure the existence of transparency requirements rather than influence itself (e.g., measuring if lobbyist registers exist, as opposed to the extent of lobbying, let alone the actual influence of lobbyists on politics). This also accentuates that while transparency is an important requirement for public integrity, transparency alone is insufficient. Strong regulations and codes of conduct are also necessary to protect political integrity [16].

Several sources have been used in public health research to analyse lobbying. Interviews are a common research method, though it is often challenging to gain access to senior policy makers, current or former corporate employees, or others willing to disclose often politically sensitive information [35]. Internal industry documents made available through the discovery process of litigation, and Freedom of Information requests have been used more recently [36, 37]. Analysis of the arguments made in policy submissions, media or other quasi-public fora are more common [38]. Publicly available data made available by governments, such as ministerial diaries or meeting records are a relatively underutilised source for public health research [20, 33, 39, 40]. In their adaptation of the CPA framework, Mialon, Swinburn [24] systematically document potential data sources for measuring political practices. Here, we build on this list of data sources by documenting a fuller range of specific data sources to measure lobbying, as well as methods available to access, extract and analyse corporate lobbying data.

## Methods

We conducted a systematic scoping review as they are useful for mapping out the evidence and identifying gaps in the literature. Following the methodological framework set out by Arskey and O’Malley [41], our review followed five steps: (1) identifying the research question; (2) identifying relevant literature; (3) screening the literature; (4) ‘charting’ the data; and (5) summarising and reporting the results. Our aim was: to identify what datasets and methods have been used to systematically analyse the extent and nature of lobbying activities globally. In recognition of the significant contribution that NGOs working in this area have made to this topic, our scoping review included both peer-reviewed and grey literature.

### Search strategies

**Fig. 1:**
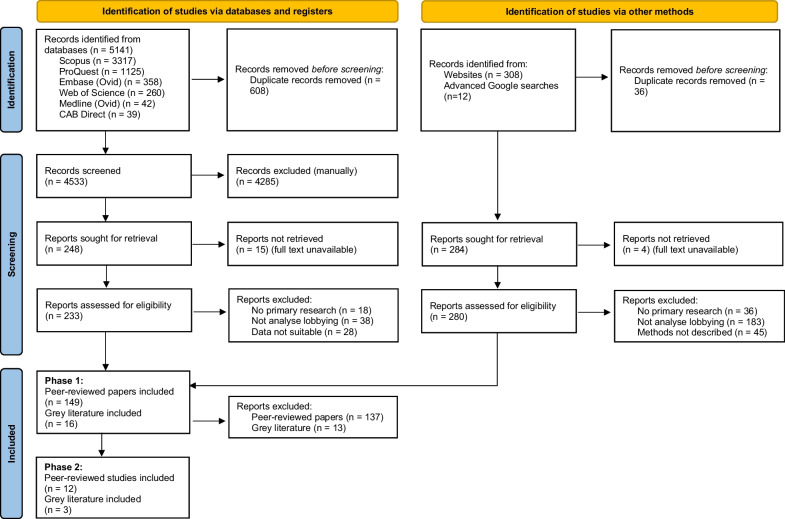
PRISMA flow diagram

Our search strategies were designed with two goals. First, we were especially interested in one mode of lobbying in particular—meetings with government employees—as these are considered the ‘gold standard’ of political access, and are especially challenging to research [42]. Second, we wanted to identify publicly available datasets about lobbying, such as government transparency registers. With these aims, JLN and KC developed a set of search terms comprising two conceptual categories: lobbying and lobbying dataset (for example transparency register or lobbyist disclosure). With the support of a health librarian, JLN completed searches for these terms across six databases: Scopus, Medline, Web of Science, Embase, CAB Direct and ProQuest. Searches were tailored to meet database requirements and limited to titles, abstracts and key words, as broader searches yielded irrelevant results. Our search strategy for Web of Science was: TS = (Lobb* OR “interest group*” OR “pressure group*” OR “outside group*” OR advoc*) AND (((TS = ((lobby* NEAR/5 disclosure*) OR (lobby* NEAR/5 regist*) OR (lobby* NEAR/5 record*))) OR ALL = ("transparency regist*" OR "minister* diar*" OR "official record*" OR (cabinet AND meet* AND record*) OR (minister* AND meet* AND record*) OR (Congress AND meet* AND record*) OR (politic* AND meet* AND record*) OR ( senator* AND meet* AND record*) OR (member AND meet* AND record*) OR (parliament AND meet* AND record*)))). Databases were searched on 29 September 2021. All searches were downloaded and imported into the citation management software Endnote X9 where duplicates were removed. 4533 documents (excluding duplicates) were identified in the database searches (see the PRISMA flow diagram in Fig. 1). Citations were exported to Excel for concurrent screening of titles and abstracts.

Following Godin et al.’s [28] approach to systematically analyse the grey literature, we conducted five Google Advanced searches, targeting different datasets in each and using similar search terms for the database searches. These was limited to ‘filetype:pdf’ as most relevant documents were in that format. We scanned the first 100 results for each search. We also conducted targeted website searches of international organisations who worked on issues related to lobbying and political influence. An initial list of organisations was sourced from the supplementary material of Mialon et al. [43], which listed 28 ‘institutions working on the influence of corporations on public health policy, research and practice’. This list was supplemented by our own knowledge of organisations working on this topic. Each organisation’s website was reviewed to identify whether they published reports on lobbying in English, with a final list of 11 organisations. Each website was searched using its embedded search function for the keyword ‘lobby’. Where possible, results were limited to reports only, as a preliminary review of other documents found that they did not provide sufficient information about the data or methods to warrant inclusion. 280 reports were downloaded for screening. Additional file 1: Appendix 1 contains the details of all peer-reviewed and grey literature search strategies.

### Screening and data extraction

MQ concurrently screened database titles and abstracts (*n* = 4533) against our inclusion criteria, with JLN double screening 10% (Table 2 documents our inclusion and exclusion criteria). Any discrepancies were discussed and resolved by JLN and MQ. Full texts of peer-reviewed studies (*n* = 233) and grey literature reports (*n* = 280) were downloaded for screening, with JLN double screening 10%. This resulted in 165 studies meeting our inclusion criteria. Due to the exploratory nature of this study, we sought to document as much information as feasible while ensuring a manageable scope. To do this, we undertook a two-stage process for data extraction.

**Table 2:** Inclusion and exclusion criteria

Inclusion criteria	Exclusion criteria
Published in English	Not published in English, or no English full text available
Conducted original/primary research	Commentary, editorial, policy submission, book report, annual report, political party platform or news article; presents model with no empirical data; referred to other studies without conducting original research
Focused on the *practice* of lobbying or the *quantity* of lobby firms and lobbyists. This included studies that: (1) Measured a lobbying activity undertaken to influence public servants (e.g., meetings, submissions, committee participation) (2) Measured lobbying expenses (3) Measured the population of lobby firms/lobbyists (e.g., registration counts)	Only examined mechanisms to address lobbying (e.g., disclosure requirements); only analysed the influence of lobbying (not the practice); only analysed strategies targeting the public (e.g., grassroots campaigns, community coalition building, direct mail services); only analysed media framing strategies; only measured the revolving door (movement between public and private sector); focused mainly on other political strategies, with lobbying only a minor component of the study (e.g., narrative analysis of tobacco industry political strategies)
Used publicly available and replicable data (including FOIs subsequently shared in public repositories)	Data sources not public or easily replicable (e.g., investigative journalism, interviews, surveys, participant observation)
Methods provide reasonable detail regarding data sources and steps taken to access, clean and analyse the data	Methods unexplained, or not described in reasonable detail to enable replication (e.g., “data analysed by author”)

For the first stage, we collected two pieces of information from all included studies (*n* = 165): data sources and indicators used to measure lobbying. We documented all publicly available data sources that were documented in the methods, including data from Freedom of Information requests that were subsequently uploaded to a public repository. We believe that this will serve as an important resource for future research on this topic. We also sought to describe the different ways that lobbying was analysed in the literature. To do this, we developed a set of ‘indicators’ used to measure lobbying activity. These were inductively developed and iteratively refined as we screened the papers, with a final set of six categories (Table 3). These categories were applied to the 165 studies included in the first stage. Some studies, especially those from the grey literature, used a combination of indicators (e.g., registrations and expenditure), in which case a primary indicator was selected for coding based on the overall focus of the study, as coding for multiple indicators was beyond the scope of the study. If meetings were one of the indicators, they were prioritised as our main interest.

**Table 3:** Indicators used to analyse lobbying activity

Indicator	Description
Registration	Count of registered lobby firms or lobbyists (often used to measure the density of lobbying populations or as a proxy for activity)
Expenditure	Amount of money spent on lobbying (e.g., firm or client expenditure, distinct from political contributions)
Meetings	Face-to-face meetings with public servants (e.g., elected officials, staff, bureaucrats); requests for meetings; reports of government branch(es), agency(s) or department(s) contacted; informal meetings
Comments	Text of written letters, submissions, comments, responses to consultations, etc
Bills	Number of bills lobbied; number of groups that lobby a bill
Committees	Participation in committee hearings/consultations; committee membership; Congressional testimony

For the second stage, data extraction was limited to studies that focused on meetings (*n* = 15) to ensure a manageable scope for analysis. As noted earlier, our primary interest was in research that analysed meetings, as they are considered the ‘gold standard’ and especially hard to research. We identified 12 peer-reviewed studies and 3 grey literature reports that we charted in Excel. Data was extracted under the following categories: article details (authors, year, title, journal, conflict of interest statement); topic (research question; main findings); location (country, state); government details (level, department, position, categorisation framework); policy details (categorisation framework); lobbyist details (industry sector, actor categorisation framework); data (sources, time period, quantity); lobbying purpose (if measured, level of detail); and challenges discussed. By applying this two-stage data extraction process, we were able to comprehensively examine what aspects of lobbying have been empirically analysed and the datasets used, while also ensuring that data extraction was feasible.

## Results

### Stage 1: lobbying indicators and datasets

**Fig. 2:**
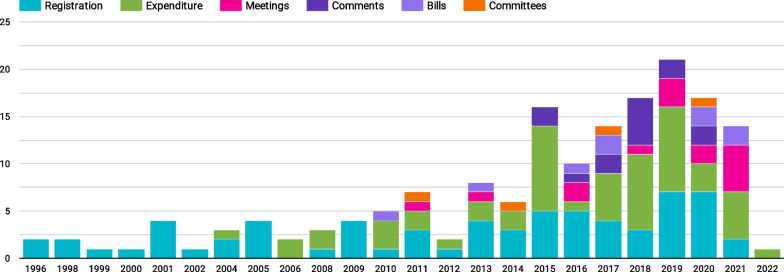
Timeline of published literature on lobbying 1996–2022

Stage 1 of our analysis found that the most common indicators used to measure lobbying activity were registrations of active lobbyists (*n* = 67) and expenditure on lobbying (*n* = 56), followed by meetings (*n* = 15), written comments and submissions made to government consultations (*n* = 14), bills (*n* = 9) and committee participation (*n* = 4). This pattern was consistent for both the peer-reviewed and grey literature reports. When examining the studies over time, lobbyist registrations and expenditures were also the earliest type of activity measured, and registrations were the only form of lobbying measured in our dataset before 2004 (Fig. 2). The registration and expenditure indicators were used in two different ways. In some cases, measuring lobbying registrations or expenditure was the aim (e.g., measuring the population of lobbyists or lobby firms, or the amount spent on lobbying). In other cases, these indicators were used as a proxy measure for lobbying activity (e.g., Baumgartner and Leech’s 2001 study [44]), as more direct measures, such as the number of meetings or hours spent lobbying, were not available in the dataset. Many of the early studies of lobbyist registrations were conducted by Gray and Lowery’s team (or drew on their data). These studies analysed the density and diversity of the USA lobbying population, and preceded the creation of consolidated government registers, instead requiring labour-intensive manual collection and coding of data from individual USA states [45].

A range of different data sources were used to analyse lobbying, including from governments, not-for-profits and commercial sources. Table 4 list the datasets identified in phase 1, which we classified as government data, publicly available data and commercially available data. We have reviewed the websites of all non-government data sources to establish whether they were available free of charge. Where unsure, we have listed them as commercially available. While we categorise many data sources as paid, we should note that researchers often have free access to these sources through their university libraries. However, cost would present a significant barrier for other organisations, such as advocacy groups to access these data. Some datasets have changed their name or no longer exist—where possible we list the current name of the source.

**Table 4:** Data sources to analyse lobbying

Government data	Publicly available data	Commercially available data
*United States* Book of the States California Secretary of State's lobbying report Eye on Lobbying (formerly Eye on Wisconsin via Wisconsin Ethics Commission's Lobbying) Federal Election Commission Foreign Agents Registration Act Harmonized Tariff Schedule of the United States IRS Form 990 Lobbying Disclosure Act (LDA) Reports Minnesota Campaign Finance Board Office of Administrative Law (California) Office of Information and Regulatory Affairs Office of Management and Budget reviews Official California Legislative Information SAM.gov (formerly Federal Business Opportunities database) Securities and Exchange Commission (comment letters, meetings, rulemaking, citations) Senate Finance Committee Social Security Administration Baby Names database Trade Advisory Committee Membership U.S. Census Bureau U.S. Department of Commerce US Congressional Hearings USA state lobbyist registers (e.g., Florida Commission on Ethics; Wisconsin Government Accountability Board, North Carolina Secretary of State) *European Union* EUR-Lex (EU) Eurobarometer surveys European Commission (Calendars of Commissioners and cabinet; meetings; Directorate-General for Budget; Consultations; Register of Expert Groups; Register of Commission Documents; Eurostat) European Parliament (Public Register of Documents; door pass register) Lobby Transparency (European Parliament Member pages via Transparency International LobbyCal plugin) Transparency Register (of the European Commission, formerly CONECCS and Register of Interest Representatives) Register of Lobbying (Ireland) electionsireland.org (Ireland) Houses of the Oireachtas (Ireland) German Lobby Register (via Bundesanzeiger, publication of the German Ministry of Justice) (Germany) OECKL Handbuch des Öffentlichen Lebens Deutschland (Germany) Le conseil national de la vie associative (France) *Other* History of the Law (via Library of the National Congress Chile, www.congreso.cl) (Chile) Lobbying Contact Reports of British Conservative Party (UK)) Public Sector Transparency Board (UK) Register for All-Party Parliamentary Groups (UK) Registry of Lobbyists (Canada) Meetings and correspondence on healthy eating (Health Canada) Government Register of Lobbyists (Australia) Productivity Commission (Australia)	Center for Progressive Reform Comparative Agendas Project (formerly Policy Agenda's project) Environmental Working Group European Patent Register European Union Policy Agendas Project codebook FollowTheMoney.org (National Institute of Money in State Politics, now merged with Open Secrets) Forbes Global 2000 Fortune 100 Lists Fortune Global 500 Freedom-of-Information request (e.g., https://corporateeurope.org/en/financial-lobby/2017/10/open-doors-forces-finance) GovTrack.us http://lobbyfacts.eu http://openinterests.eu https://www.voteview.com/ Interarena project INTEREURO project International Standard Industrial Classification scheme Investor Responsibility Research Center Italian Stock Exchange LinkedIn LobbyView Open Secrets (formerly Center for Responsive Politics) Public Citizen Sunlight Foundation The European Election Studies UN Accreditation database Vote Smart Voter Information Services	American lobbyists directory BoardEx Breach Level Index Compustat Congressional Districts in the 1990s: A Portrait of America Directory of British Associations Directory of Corporate Affiliations Encyclopedia of Associations First Street Guida Monaci sul Sistema Italia (Italian interest group registration) International Accounting Standards Board LexisNexis Nealon’s Guide Orbis Pyttersen Almanak, Netherlands Refinitiv Datastream Russell 2000 Index SDC VentureXpert State Full Text of Bills database on Nexis Academic Universe The European Public Affairs Directory Thomson Financial Securities Data Thomson Reuters Business Classification Thomson Reuters Stock Ownership Washington Representatives (formerly www.lobbyists.info, now https://www.washingtonrepresentatives.com/) WestLaw Next Yearbook of International Organizations

The most frequently used datasets were the US-based Open Secrets database (created by the Center for Responsive Politics, which merged in 2021 with the National Institute of Money in Politics) and the European Transparency Register of the European Commission. In addition to government data, most studies also drew on other datasets to augment and interpret the data. Several studies drew on the Comparative Agendas Project (formerly the Policy Agendas Project) to analyse the different issues that were the target of lobbying [46, 47]. Other studies used business databases, such as Compustat or BoardEx, to analyse commercial attributes including industry sector, revenue, parent company or board membership [48, 49].

Due to most datasets focusing on a single political jurisdiction, few studies were comparative. Indeed, approximately two-thirds of the 165 studies we screened were based in the USA (*n* = 113). While many studies drew on different data sources, the majority of research on lobbying expenditure used the Open Secrets database (48 of 56 studies), also meaning that most research on lobbying expenditure has focused on the United States.

### Stage 2: studies analysing lobbyist meetings

Stage 2 of our analysis found that few studies included in our review (*n* = 15) systematically analysed meetings between lobbyists and government officials, legislatures or their staff. However, this number underestimates the attention to meetings in the literature overall, in particular in the original grey literature reports that we screened, as many of these were excluded from our analysis as the lacked sufficiently detailed methods. Table 5 presents a summary of key findings.

**Table 5:** Studies analysing lobbyist meetings

Lead author	Title (short)	Year	Journal	Jurisdiction	Govt. level	Govt. Department	Govt. actors mentioned	Industry sector	Specific activity measured	Lobbying data sources	Other data sources	Time period	Volume of data	Lobbying purpose analysed	Level of detail about purpose
McKay	The decision to lobby bureaucrats	2011	Public Choice	US	National	Many	–	–	Contact made with government	Washington Representatives data; Lobbying Disclosure dataset created by Baumgartner & Leech (2001)	–	1996	3362 lobbyist-issues; 19,309 lobbyist reports	N	n/a
Boehmke	Business as usual	2013	Journal of Public Policy	US	National, State	Many	–	–	Contact made with government branches concerning specific issues	LDA reports; lobbying reports from Minnesota state's Campaign Finance Regulation website	–	1996, 2004	1,092 organisations; 5,935 lobbyist-issue-organisation observations; 3,975 issue-organisation observations (state); 5838 groups; 49,518 total issues (Federal)	N	n/a
ALTER-EU	National Representations in Brussels	2016	ALTER-EU	EU	Supranational (member state permanent representations to the EU)	Member state permanent representations to the EU	Permanent Representatives; Deputy Permanent Representatives	–	Meetings	Access to Information requests provided by Ireland, Romania, the Netherlands, and Poland	EU’s Joint Transparency Register	2014–2015	267 lines	Y	Topic of discussion listed, but not position of interest group
Reyes	Carbon Welfare	2016	Corporate Europe Observatory	EU	Supranational (EU parliament and council)	Climate; Energy	Commissioners for climate and energy; Directorates General Clima (ClimateAction); Directorates General Growth; Energy Intensive Industries expert group; European Parliamentarians	Climate	Meetings, letters, position papers, evens (e.g., dinners, cocktail parties, birthday parties)	Open calendars of European Commissioners and their cabinets; European Commission Transparency Register	LobbyFacts database	2014–2016	–	Y	Moderately detailed; illustrative examples presented
Boucher	Who you know in the PMO	2018	Canadian Public Administration	Canada	National	Many	Civil servants; politicians	–	Oral and arranged communications with designated public office holders	Communication reports filed in the Canadian lobbying registry	–	2008–2013	72,082 lobbying contacts; 1,804 lobbying organizations	N	n/a
Coen	Between cheap talk and epistocracy	2019	Public Administration	EU	Supranational (European Parliament)	Committee on economic & financial affairs (ECON); Committee on internal market & consumer protection (IMCO); Committee on civil liberties, justice & home affairs (LIBE)	Members of European Parliament	–	Attendance in a EP hearing	EP’s Joint Transparency Register; EP’s online search engine	–	2009–2014	74 hearings; 357 speakers	N	n/a
Boucher	Consultant Lobbyists and Public Officials	2019	Political Studies Review	Canada	National	Many	Ministers; partisan advisors (exempt staff); public servants (deputy and assistant deputy ministers)	–	Contact a public office holder	Canadian Lobbyist Registry	–	2008–2016	1533 consultant lobbyists; 23,525 communication reports; 2235 different public offic holders (communicated with lobbyists)	N	n/a
Cullerton	Doctors rule	2019	International Journal of Environmental Research and Public Health	Australia	State (NSW, QLD)	Health	Ministers	Food	Meeting with state minister	Government websites	–	2013–2018	5025 diary entries	Y	Topics related to nutrition listed, but little detail provided (e.g., junk food advertising, National Nutrition Week, childhood obesity)
Alves	Corporate political strategies in Europe	2020	Business and Politics	EU	Supranational (European Commission)	Many	Commissioners; commissioners' cabinet members; directors-general	Energy, financials, technology, telecommunication services, and utilities	Meetings	EC Transparency Register; information about Commission meetings	Thomson Reuters Business Classification; Fortune Global 500; Forbes World's Biggest Public Companies	2014–2016	1,845 companies	N	n/a
Haeder	Out of the public’s eye?	2020	Interest Groups & Advocacy	US	National (Office of Management and Budget)	Many	US President’s Office of Information and Regulatory Affairs' (OIRA) presidential appointee; white house officials; representatives from government agencies; members of congress	–	Oral communications (meetings, phone calls)	White House Web site	Unified Agenda; Federal Register; Open Secrets; draft-Final Rule text	2005–2011	786 meetings; 4264 attendees; 315 distinct rules	N	n/a
Liu	Campaign Contributions and Access to Congressional Offices	2021	Political Research Quarterly	US	National	Many	–	–	Meetings	Foreign Agent Registration Act data via Department of Justice (https://efile.fara.gov/ords/f?p=107:21:::NO:::; revolving door profiles from CRP)	DW-NOMINATE (now VoteView https://voteview.com/) documenting member ideology; CRP: campaign contibutions from lobbyists and PACs affiliated with lobby firms; revolving door data from Center for Responsive Politics	1998–2019	981 lobby firms; 9,498 lobbyists; 5,357 “supplemental reports” comprising more than 70,000 records of lobbyist seeking contact; lobbyist campaign contributions (1430 in both datasets; 13,484 in CRP records only; 10,910 in FARA reports only)	Y	Author notes data not consistent enough for inclusion in statistical analysis
Huwyler	Interest group tactics and legislative behaviour	2021	Journal of European Public Policy	Ireland	National	Many	Legislators	–	Meetings, Lobby days, Events/receptions, Informal communication, Virtual meetings, Phone calls, Conference calls, Letters, E-mails, Submissions, Social media	Irish Register of Lobbying	Houses of the Oireachtas Open Data APIs (oral and written parliamentary questions); Nealon’s Guide; electionsireland.org; wikipedia.org	2015–2019	217,886 lobbying attempts; 67,347 parliamentary questions tabled by Irish legislators	N	n/a (although statement about 'intended results' part of lobbyist return data)
Coen	Lobbying Brexit Negotiations	2021	Politics and Governance	EU	Supranational (European Commission)	Many	EU's Chief Negotiator and Brexit Task Force	–	Meetings	European Commission website	EU’s Joint Transparency Register	2016–2020	159 participants;113 interest groups	Y	Two general categories used
Mulligan	Stakeholder interactions with the federal government related to Bill S-228 and marketing to kids in Canada	2021	CMAJ open	Canada	National	Health Canada	Parliamentarians and their staff; Prime Minister’s Office; Ministers and parliamentary secretaries; Ministerial staff; Members of Parliament, Senators and their staff; Civil servants; Privy Council Office; Deputy ministers; Assistant deputy ministers; Other government officials	–	Meetings; correspondence; documents	Health Canada’s “Meetings and correspondence on healthy eating” database; Registry of Lobbyists	–	2016–2019	139 meetings; 65 lobbying registrants; 215 lobbying registrations; 3418 communications	N	n/a
Influence Map	The Battle for Ambitious EV policy in the UK	2021	Influence Map	UK	National	–	–	Automotive (energy briefly mentioned)	Meetings; submissions; public comments	UK government’s transparency platform	Submissions; public statements	2017–2020	436 meetings	Y	Significant detail of interest group positions (based on non-meeting data sources, e.g. public comments)

All studies were in high-income contexts: the European Union (*n* = 5), the United States (*n* = 4), Canada (*n* = 3), Australia (*n* = 1), Ireland (*n* = 1) and the United Kingdom (*n* = 1). 13 studies focused at the national or supranational (e.g., European Commission) level, with one study comparing national and state-level data, and another study comparing two states.

Seven studies declared that they had no conflict of interest [39, 55,50-]. The others did not make a declaration.

The studies explored different questions. Some analysed which types of interest groups lobbied most frequently [39, 51, 60,55-]. Others analysed the characteristics of lobbyists to explain their access to government officials. For example, Boucher [50] used empirical data to show how the professional background of lobbyists as either a well-connected generalist (e.g., with revolving door background) or an issue expert influenced whether they contacted political or bureaucratic public office holders. Similarly, Alves [61] found that lobbyists with political knowledge had greater access to high-level officials within the European Commission. Liu [54] found that lobbyists made political donations not usually to build relationships, but rather to maintain existing ones. Huwyler [53] drew on the extensive data disclosed in the Irish Register of lobbying to show that synchronous, face-to-face lobbying strategies (e.g., meetings, events) are more effective than asynchronous strategies (e.g., e-mails, social media). McKay [62] analysed the factors determining whether interest groups lobby the bureaucracy as opposed to the legislature, finding that conflict leads to both being lobbied more, whereas the bureaucracy is lobbied more if the issues concern only a small number of parties or if the issues have a long life-span.

Only five studies analysed the purpose of lobbying (including all three grey reports) [39, 55, 60,58-]. The data presented by the grey literature reports was sourced from a much wider range of documentary sources than the peer-reviewed literature, and much of the information presented about the meeting purpose came from those additional sources. The two peer-reviewed studies that presented data about the purpose of the meetings could only access very general information about the topic of the meeting, and no detailed information about the intention of the interest group in relation to that topic.

Different approaches were used to analyse three key variables: the types of government actors targeted by lobbying; the policies of interest; and the lobbyists and/or their clients. The most common variable considered for government actors was whether they were part of the legislative/elected branch of government or whether they were part of the bureaucracy. Six studies made this distinction, noting that depending on the political system, these actors played different roles in terms of setting agendas, policy making, designing rules and regulations, and thus have different interests and incentives [50-52, 54, 62, 63]. Three studies differentiated between specific positions and roles in government, with Mulligan [51] ranking positions withing the Canadian government according to their perceived importance and influence. Finally, some studies looked at the role of political party affiliation and leadership, and whether the public official was a member of an influential committee (e.g., the House Ways and Means committee in the US).

Some studies classified the policy domains. Four papers focused on case studies of a particular policy issues (nutrition, climate change, marketing to children) [39, 51, 58, 60]. Other studies sought to classify a wide range of topics, either using the policy categories used by the registers (of which there were 46 categories used in Canada, 96 in Minnesota and 76 in the USA federal register), or proposing a new classification scheme. What is relevant to note here is the inconsistencies in how policies are classified, both across different jurisdictions (e.g., the USA and the EU), as well as across studies. For example, one framework grouped Health, Education and Social Affairs as three separate categories, while another framework grouped them as “Health, education and social policy” [52, 53]. We will return to this challenge for conducting comparative research in the discussion.

The most similar frameworks related to the types of organisations engaged in lobbying. Table 6 lists the most common categories used to classify groups. These categories focused primarily on the type of actor, rather than the industry sector of the client hiring the lobbyists. Only two studies differentiated between industry sectors, both of which developed their own categorisation frameworks [39, 59].

**Table 6:** Categories used to classify actors engaged in lobbying

Category	Other terms
Business	Company, Private firm, Corporate bodies, For-profit organisations
Business association	Industry group, Peak body, Trade group, Trade association
Union	Labour union, Trade union
Research	Research institutions, Academic organisation, University
Public interest	Advocacy, Civil society group, Public interest group, Public interest association, Non-governmental organization, Charity, Citizen groups, Public institution
Government	State government, Local government, Government organisations, Federal agencies, Foreign governments, Public bodies
Identity group	Religious, Hobby group
Consultancy	Professional consultancies, consultants
Think tank	
Law firm	
Lobbying firms	
Individuals	

All studies noted challenges regarding the data. Two overarching themes emerged from these challenges. The first and most common challenge related to deficiencies in the data, where information for understanding the purpose and influence of lobbying was not provided. For example, Boucher [50, 52] noted that the Canadian data on lobbying contacts did not contain information about the motivations of the lobbyists or the government officials that lobbyists contact, nor did the register provide information about lobbying expenditure. Several studies noted that not all actors that engage in lobbying are included within registers, such as in-house lobbyists employed directly by companies or trade associations. Similarly, not all those contacted by lobbyists are included with the registers. Interactions are often only recorded for high-level, or elected officials, whereas interactions with advisers, staff members or the bureaucracy are not always recorded. Inconsistency and incompleteness in the data was a common concern, with papers noting that some elements of the disclosure are seen as voluntary or optional [54, 61].

The second theme related to the format and quality of the data. Challenges here included that data were non-downloadable [55], data lacked unique identifiers, making it challenging to match lobbyists or clients [57], and that data were only available as PDFs in unstandardised formats [56]. To overcome these challenges, time-consuming cleaning and coding of the data was required. In some cases, this was done manually, however in others data science and machine learning tools were used to automate steps. For example, Haeder and Yackee [57] used plagiarism detection software to compare the text of draft and final regulations in the US, and Liu [54] used machine learning models to classify lobbyist contacts as either Congress members or their staff. These examples illustrate some of the options to overcome the challenges inherent in working with patchy and poor-quality data.

## Discussion

A tremendous body of work analyses lobbyists, their clients and their activities. One of the most common themes across virtually every paper was the challenge of researching lobbying activity due to the lack of critical data. This is seen in the limited geographic scope of the studies included here, with the vast majority based in the US, the EU and Canada. Research on lobbying is largely limited by what governments have chosen to make available. For this reason, lobbying activity was most often measured through proxies—registrations or expenditures. Similarly, most of the studies we analysed about lobbyist meetings focused on two questions. First, who were the groups with the greatest access to governments (mapping the population of organisations engaged in lobbying). And second, what were the attributes of the lobbyists that enabled them to gain access to government representatives, their staff, or the bureaucracy. Notably absent was a focus on what the meetings were about, or the position that the lobbyist supported, as this was not consistently disclosed or disclosed in such general terms that it provided few relevant insights. The one exception to this was the report from Influence Map [58], however this information came from secondary sources, not from meeting records.

The relatively recent creation of national transparency registers has made it far easier to research lobbying, though this is still dependent on specific disclosure requirements of the register. Research projects preceding the registers often required pain-staking manual collection and organisation of lobbyist data (e.g., Gray and Lowery’s [64] seminal analysis of USA state lobbyists), leading to long delays between the lobbying activity and the ability to analyse it, significant gaps in the record as well as an increased likelihood of human error [65, 66]. In some cases, other sources provide a more complete picture of lobbying than the official lobbyist register, such as Health Canada’s database of meetings and correspondence [40]. This review has emphasised the inconsistency in the scope and quality of data made available by governments, in particular in countries outside North America and the EU, underscoring the need to improve transparency requirements to ensure that the data exist. The OECD’s report *Lobbying in the 21st Century* systematically audited the lobbying disclosure requirements of 41 countries, finding only 23 had requirements in place. Clearly, much could be done to increase the availability and consistency of information on lobbying internationally.

This review has highlighted a range of opportunities for public health actors to learn from political science research on lobbying, which we elaborate on briefly. A first learning is simply expanding awareness of the potential data sources that can be analysed, and the indicators that could be used to measure the extent of lobbying. We have taken an international approach, which builds on previous work mapping out the availability of US-focused datasets [67]. Many public health studies have conducted rich case studies analysing interactions between lobbyists and politicians based on interviews with policy makers and documenting the conflicts of interest and influence this has on policy making [21, 68, 69]. Complementing these case studies with large n studies can help to contextualise these findings within the broader universe of lobbying. It can also provide a more objective measure of influence (and possibly triangulation), as interviews with elites may result in them exaggerating or minimising information to suit their agenda [70]. Data on lobbying can encompass many elements, including spending on lobbying, the makeup of lobbying organisations, the networks of interest groups, the political views of lobbyists, as well as the various forms that lobbying can take, including meeting with ministers, advisors and bureaucrats, requests for meetings, submissions, participation in committees and more [65]. From a practical standpoint, the range of different datasets available to analyse lobbying may prove useful for efforts seeking to monitor commercial determinants of health [71, 72]. Further, while many of the datasets documented here exist in the Global North, similar datasets may exist in other countries which we are not aware of. The datasets identified here could be used to guide further research scoping of the range and quality of datasets available in other countries.

A second learning concerns opportunities to augment lobbying data. Our review identified several creative examples of working around data limitations, such as freedom of information requests used to access more complete datasets [60], and linking government data with external datasets, such as Thomson Reuters Business Classification and the Fortune Global 500 [61]. There are other examples in the literature, with the Wayback Machine used to access earlier versions of registers [73]. In some cases, the government agencies or organisations providing the data had more information available on request (e.g. the specific dates of door registration passes) [74]. Many of the studies in our initial sample analysed business datasets or proprietary data on lobbyists and linked these with the publicly available data provided by governments. The MIT research project LobbyView, for instance, augmented the US lobbyist disclosure data with client data from Compustat [75]. Linking these different data sources together offers a greatly enriched dataset and opportunities to analyse whether different commercial attributes influence lobbying behaviours. For instance, the LobbyView database was used to analyse efforts to influence USA policy towards the WHO [76]. To capitalise on these opportunities, public health organisations could invest in accessing and training researchers in the use of these business databases.

A third learning is that political science scholarship helps to interpret and explain lobbying activities. Several of the studies in our review focused on the attributes of lobbyists as an explanatory variable, drawing on LaPira and Thomas’ [77] analysis of the different resources that lobbyists bring to the table. They describe two lobbyist architypes—the ‘librarian’ and ‘K Street Kingpin’—who offer technical expertise and political access, respectively. Differentiating between the attributes of lobbyists can assist public health researchers to better understand the nature of lobbying, what circumstance may lead to lobbyists with different expertise engaging with governments and their influence on policy outcomes. It highlights the importance of deeply scrutinising the revolving door, as evidence shows that these lobbyists are most frequently employed by businesses and are the most active and influential [77]. To explain which issues lobbyists are interested in, studies have linked lobbyist data with the USA Policy Agendas Project coding framework, which systematically classified USA congressional hearings to map out which policies had the attention of the government over time [78, 79]. This codebook was subsequently adapted to other political jurisdictions and used to create the Comparative Agendas Project’s Master Codebook [80]. Drawing on this lobbying research will complement the increasing and much needed engagement of public health scholarship with political science scholarship [17].

Fourth, our review of research on lobbying also accentuates the need to improve the quality of lobbying transparency in terms of both the content and format. For instance, the data sources we documented in Table 4 provide examples of existing datasets that may include information missing from others. This could complement several ongoing initiatives to improve lobbying transparency, including the OECD’s update of its public integrity principles and Transparency International’s analysis of public integrity datasets [81]. These initiatives offer useful examples of what good practice looks like to which governments could aspire. Monitoring lobbying, and corporate political activities more generally, has been identified as a key obligation for governments [82].

Lobbying data can also be improved in terms of *how* the data are provided. Several of the studies analysed discussed the practical challenges of working with the data, and the limitations this presented for analysis. A common challenge across both the studies in our review and the wider literature on lobbying is the need to clean the data to match names that have been entered differently [33]. Requiring a unique identifier, for example, would help to address this challenge and make the data more easily searchable. While this sounds straightforward in theory (e.g., assign each commercial entity or lobbyist a unique ID when they register, and likewise for public servants), in practice it presents several difficulties. Who would be responsible for implementing and enforcing this? If the lobbyist works for a multinational company and visits officials around the world, how do we ensure the unique identifier is consistent and linkable across countries? Moreover, the provision of unique identifiers would require significant political commitment to integrity which few countries internationally have so far demonstrated. Despite these hurdles, the benefits for analysis and monitoring are manyfold. For instance, unique identifiers would enable the linking of disparate datasets, for example linking lobbyist registers with business databases such as Compustat (as many of the studies did) or linking datasets across jurisdictions to compare lobbying behaviours. Unique identifiers would also greatly aid research and monitoring of the revolving door, as the ID could follow public servants if they move into the private sector and vice versa. Other recommendations to improve the functionality and ‘openness’ of the data have been thoroughly documented elsewhere [81, 83, 84].

Fifth, and finally, this research identifies possible allies for public health advocacy and research collaborations. The diversity of issues and topics in the literature reveals the alignment between public health interests in CDoH and those interested in public integrity, corporate accountability whose work has focused on other domains, e.g., human rights abuses, climate change or political corruption [85]. One example of this alignment is the Global Data Barometer, launched in July 2022 [81]. Created by the Open Government Partnership and Transparency International, the Political Integrity module collects data on five dimensions: political party finance, political interest declarations, lobbying registers, public consultation in rule-making; and right-to-information. The Barometer also differentiates between two elements of data important for analysis: quality related features (the content of the data) and open data related features (regarding the useability of the data). The information collected in the Barometer is valuable from a public health perspective to ensure that that undue commercial influence is prevented. Finding common interests, such as improving public integrity and enhancing transparency, presents an opportunity to align public health interests with existing coalitions and jointly build capacity.

## Conclusions

There are many fruitful areas for further research to extend our analysis. Our study focused on only a selection of lobbying indicators, and it would be useful to document the methods used to analyse other political practices, such as campaign donations and the revolving door. Additionally, to ensure a feasible scope of analysis, our search strategy focused on the practice of lobbying, in particular meetings with public officials. A targeted search focusing on other elements of lobbying, such as policy submissions, the revolving door, the types of clients that engage lobbying services, or interest group influence more widely, is likely to identify different studies and datasets and is worth exploring in subsequent research projects. Our study was limited to publicly available and replicable data, especially lobbyist registers, however it would be useful to analyse the data uncovered through investigative journalism, interviews, ethnography or other methods more challenging to replicate. This could help to provide some of the rich details about why lobbying occurred and what was discussed in meetings, which is often missing from public repositories. While this might be less immediately translatable into efforts to systematically monitor lobbying with large studies, it can help to identify the universe of possible information, and provide rationales for improving existing public repositories so this less visible information is made public. A final observation is that many of the grey literature reports were excluded from this review for not providing sufficient detail regarding their methods, which may reflect the different (non-academic) audience. However, it also highlights an opportunity for academic researchers to collaborate with organisations working on these issues, and to support the documentation and explanation of the datasets and methods used in the reports so that they have greater rigor.

As lobbying is a challenging corporate practice to monitor, this study aimed to explore the range of potential datasets and methods available to research lobbying activities for the purposes of monitoring CDoH. By systematically reviewing the published literature, our study provides insights for researchers, advocates and policy makers as to what datasets and methods are available, how these could be applied to monitor CDoH and what changes are needed to improve the quality of political transparency and public integrity so that public health is prioritised.

## Supplementary Information

**Additional file 1: Appendix 1. **Database and grey literature search strategies.

## Data Availability

The datasets analysed during the current study are available from the corresponding author on reasonable request.
