# Supplementary material for: Aiding empirical research on the commercial determinants of health: a scoping review of datasets and methods about lobbying
Source: Health Res Policy Syst. 2023 Jun 19;21:56. doi: 10.1186/s12961-023-01011-8 (PMC10278313; doi:10.1186/s12961-023-01011-8)
Supplement: Supplementary file 1 — Additional file 1: Appendix 1. Database and grey literature search strategies. [file 12961_2023_1011_MOESM1_ESM.docx]

Appendix 1: Database and grey literature search strategies

**Category groups (and synonyms):**

| **#** | **Concept** | **Search terms** |
| --- | --- | --- |
| 1 | Lobbying | Lobb* OR “interest group*” OR “pressure group*” OR “outside group*” OR advoc* |
| 2 | Lobbying dataset | "transparency regist*" OR "minister* diar*" OR "official record*" OR (lobby* W/5 disclosure*) OR (lobby* W/5 regist*) OR (lobby* W/5 record*) OR (cabinet AND meet* AND record*) OR (minister* AND meet* AND record*) OR (Congress AND meet* AND record*) OR ( senator* AND meet* AND record*) OR (parliament AND meet* AND record*) OR (politic* AND meet* AND record*) OR (member AND meet* AND record*) |

**Database search strategies**

| **Search terms** | **Platform or URL** | **Date** | **Results** |
| --- | --- | --- | --- |
| Peer-reviewed database |  |  |  |
| ( TITLE-ABS-KEY ( lobb* OR "interest group*" OR "pressure group*" OR "outside group*" OR advoc* ) AND ALL ( ( lobby* W/5 disclosure* ) OR ( lobby* W/5 regist* ) OR ( lobby* W/5 record* ) OR "transparency regist*" OR "minister* diar*" OR "official record*" OR ( cabinet AND meet* AND record* ) OR ( minister* AND meet* AND record* ) OR ( congress AND meet* AND record* ) OR ( politic* AND meet* AND record* ) OR ( senator* AND meet* AND record* ) OR ( member AND meet* AND record* ) OR ( parliament AND meet* AND record* ) ) ) | Scopus | 29/09/2021 | 3317 |
| TS=(Lobb* OR “interest group*” OR “pressure group*” OR “outside group*” OR advoc*) AND (((TS=((lobby* NEAR/5 disclosure*) OR (lobby* NEAR/5 regist*) OR (lobby* NEAR/5 record*))) OR ALL=("transparency regist*" OR "minister* diar*" OR "official record*" OR (cabinet AND meet* AND record*) OR (minister* AND meet* AND record*) OR (Congress AND meet* AND record*) OR (politic* AND meet* AND record*) OR ( senator* AND meet* AND record*) OR (member AND meet* AND record*) OR (parliament AND meet* AND record*)))) | Web of Science | 29/09/2021 | 260 |
| (Lobb* or "interest group*" or "pressure group*" or "outside group*" or advoc*).ab. and ((lobby* adj5 disclosure*) or (lobby* adj5 regist*) or (lobby* adj5 record*) or "transparency regist*" or "minister* diar*" or "official record*" or (cabinet and meet* and record*) or (minister* and meet* and record*) or (Congress and meet* and record*) or (politic* and meet* and record*) or (senator* and meet* and record*) or (member and meet* and record*) or (parliament and meet* and record*)).af. | Embase (Ovid) | 29/09/2021 | 358 |
| (Lobb* or "interest group*" or "pressure group*" or "outside group*" or advoc*).ab. and ((lobby* adj5 disclosure*) or (lobby* adj5 regist*) or (lobby* adj5 record*) or "transparency regist*" or "minister* diar*" or "official record*" or (cabinet and meet* and record*) or (minister* and meet* and record*) or (Congress and meet* and record*) or (politic* and meet* and record*) or (senator* and meet* and record*) or (member and meet* and record*) or (parliament and meet* and record*)).af | Medline (Ovid) | 29/09/2021 | 42 |
| (ab:(Lobb* or "interest group*" or "pressure group*" or "outside group*" or advoc*) AND ((lobby* NEAR/5 disclosure*) OR (lobby* NEAR/5 regist*) OR (lobby* NEAR/5 record*) OR "transparency regist*" OR "minister* diar*" OR "official record*" OR (cabinet AND meet* AND record*) OR (minister* AND meet* AND record*) OR (Congress AND meet* AND record*) OR (politic* AND meet* AND record*) OR ( senator* AND meet* AND record*) OR (member AND meet* AND record*) OR (parliament AND meet* AND record*))) | Cab Direct/Global Health | 29/09/2021 | 39 |
| ab(Lobb* OR "interest group*" OR "pressure group*" OR "outside group*" OR advoc*) AND ((lobby* NEAR/5 disclosure*) OR (lobby* NEAR/5 regist*) OR (lobby* NEAR/5 record*) OR "transparency regist*" OR "minister* diar*" OR "official record*") AND stype.exact("Reports" OR "Scholarly Journals") | ProQuest | 07/10/2021 | 1125 |

**Grey literature searches**

Google Advanced Search

Note: filter was applied to limit to pdf documents (to restrict to reports)

Searches “All results” – first 10 pages, representing 100 results screened

| **#** | **Search** | **Date** | **# results** | **# results screened** | **# new potentially relevant records** |
| --- | --- | --- | --- | --- | --- |
| 1 | ("interest group" OR "pressure group" OR Lobby OR lobbyist OR lobbied OR advocate OR advocacy) AND "transparency register" filetype:pdf | 6/11/2021 | 13,100 | 100 | 8 |
| 2 | ("interest group" OR "pressure group" OR Lobby OR lobbyist OR lobbied OR advocate OR advocacy) AND "ministerial diaries" filetype:pdf | 6/11/2021 | 956 | 100 | 1 |
| 3 | ("interest group" OR "pressure group" OR Lobby OR lobbyist OR lobbied OR advocate OR advocacy) AND "disclosure" filetype:pdf | 6/11/2021 | ~21,600,000 | 100 | 0 |
| 4 | ("interest group" OR "pressure group" OR Lobby OR lobbyist OR lobbied OR advocate OR advocacy) AND "record" filetype:pdf | 7/11/2021 | ~124,000,000 | 100 | 2 |
| 5 | ("interest group" OR "pressure group" OR Lobby OR lobbyist OR lobbied) AND "meeting" filetype:pdf | 7/11/2021 | ~21,400,000 | 100 | 1 |

Targeted website searches

An initial list of organisations was sourced from the supplementary material of the paper: (Mialon, M., Vandevijvere, S., Carriedo-Lutzenkirchen, A., Bero, L., Gomes, F., Petticrew, M., ... & Sacks, G. (2020). Mechanisms for addressing and managing the influence of corporations on public health policy, research and practice: a scoping review. *BMJ open*, *10*(7), e034082.), which listed 28 ‘institutions working on the influence of corporations on public health policy, research and practice’. Each of the websites (if available in English) was searched for the term ‘lobby’ to identify whether the organisation published research (e.g. reports) on the topic. For each included website, all reports were searched (e.g. limit to publications, reports to ensure a research focus vs a news report). Where possible, a keyword search ‘lobby’ is used to further filter the results.

| **Organisation** | **Website** | **Date** | **Search** | **# results** | **# results screened** | **# new potentially relevant records** |
| --- | --- | --- | --- | --- | --- | --- |
| 1 | Alliance for Lobbying Transparency and Ethics Regulation (ALTER-EU) | https://www.alter-eu.org/documents?taxonomy_vocabulary_4_tid=All&field_date_publication_  value%5Bvalue%5D%5Bdate%5D=&taxonomy_vocabulary_1_tid=2 | All reports | 21 | 21 | 3 |
| 2 | Centre for Research on Multinational Corporations | https://www.somo.nl/research/ | Lobby (limit to publications) | 33 | 33 | 0 |
| 3 | Open Secrets | https://www.opensecrets.org/news/reports/ | All reports | 27 | 27 | 8 |
| 4 | CorpWatch | https://www.corpwatch.org/explore/reports | All reports | 14 | 14 | 1 |
| 5 | Corporate Accountability | https://www.corporateaccountability.org/resources/type/reports/#filter | All reports | 12 | 12 | 0 |
| 6 | Corporate Europe Observatory (CEO) | https://corporateeurope.org/en/reports | All reports | 50 | 50 | 9 |
| 7 | InfluenceMap | https://influencemap.org/reports/Reports | All reports | 18 | 18 | 2 |
| 8 | Friends of the Earth | https://www.foei.org/?s=lobby | Lobby | 13 | 13 | 0 |
| 9 | Multinationals Observatory | https://multinationales.org/spip.php?page=recherche&lang=en&recherche=  lobby&rubrique[]=Investigations | Lobby, limit to investigations | 37 | 37 | 1 |
| 10 | Transnational Institute | https://www.tni.org/en/search/language/en/types/Paper/types/Publication/  types/Report?search=lobby | Lobby, limit to papers, publications, reports | 73 | 73 | 1 |
| 11 | Transparency International | https://www.transparency.org/en/search/advanced?CRAFT_CSRF_TOKEN=uzSUdkeOtYVUdvdS71iYYINN9UOjDpmeoCPUBVDL0ViMv8QNL58cYtRb9yUY1oLnITiVOIA-qyzmApYg8mDs8_MTn0A7rosXz8WlQnv3elU%3D&query=lobby&type=publication&country=*&priority=*&advocacy=*&order=score | Advanced search, Lobby, limit to publications | 10 | 10 | 2 |
